# Eilat virus isolated from *Culex univittatus* mosquitoes from the Namibian Zambezi Region influences *in vitro* superinfection with alpha- and flaviviruses in a virus-species-dependent manner

**DOI:** 10.1371/journal.pone.0312182

**Published:** 2024-12-20

**Authors:** Heiko D. Guggemos, Anne Kopp, Katrin Voigt, Matthias Fendt, Selina L. Graff, John K. E. Mfune, Christian Borgemeister, Sandra Junglen

**Affiliations:** 1 Institute of Virology, Charité - Universitätsmedizin Berlin, Corporate Member of Free University Berlin, Humboldt-University Berlin, and Berlin Institute of Health, Berlin, Germany; 2 Department of Environmental Science, University of Namibia, Windhoek, Namibia; 3 Center for Development Research (ZEF), University of Bonn, Bonn, Germany; The University of Queensland, AUSTRALIA

## Abstract

The genus *Alphavirus* harbors arboviruses of great concern, such as the Chikungunya virus and the equine encephalitis viruses. Transmission of pathogenic alphaviruses by mosquitoes could be influenced by insect-specific alphaviruses such as Eilat virus (EILV). However, insect-specific alphaviruses are rarely found in wild mosquitoes and only a few have been described in the literature. Here, we report the detection of EILV in a *Culex univittatus* mosquito from the north-eastern Namibian Zambezi region. Full genome analysis of MP458-NA-2018 showed 94.5% nucleotide identity to an EILV isolate from Israel. MP458-NA-2018 grouped with EILV in phylogenetic analysis and was placed within the clade of insect-specific alphaviruses. The virus was isolated in mosquito cells and shown to be restricted to insects as hosts by the inability to infect different vertebrate cell lines and a complete block of virus replication at 34°C. We further showed that infection of cells with EILV MP458-NA-2018 reduced production of infectious particles of Sindbis virus by 2000-fold over the entire course of infection, whereas reduction rates of Bagaza and Middleburg virus were approximately 3-10-fold and dependent on time after infection. While production of infectious particles of cells superinfected with the Chikungunya virus were approximately 30-fold reduced and more pronounced at a lower multiplicity of infection of 0.01, EILV seemed to enhance production of West Nile virus infectious particles by >5-fold when superinfected at a multiplicitiy of infection of 0.1. In summary, EILV from the Namibian Zambezi region influences *in vitro* replication of endemic flavi- and alphaviruses.

## Introduction

The genus *Alphavirus* comprises 32 species and is the only genus in the family *Togaviridae* [1]. Alphaviruses are divided into a diverse group that is transmitted by arthropods and infects a wide range of vertebrates ranging from mammals to birds and fish [2] and a less diverse group of viruses with a host range restricted to insects [3]. Alphaviruses are found on all continents [4]. The genus contains several important human and animal pathogenic viruses, like the Chikungunya virus (CHIKV), Sindbis virus (SINV) and eastern equine encephalitis virus (EEEV) [5]. Alphaviruses form enveloped virions of spherical shape with a diameter of around 65 to 70 nm [6]. Their genome is composed of single-stranded positive-sense RNA, approximately 11,000 to 12,000 nucleotides in length, with a 5’ cap structure and 3’ poly(A) tail, which is coding for four non-structural (NSP1-NSP4) and six structural proteins (SPs) (Capsid, E3, E2, 6K/TF, and E1) [6].

Eilat virus (EILV) was the first insect-specific alphavirus to be described, found in a pool of *Anopheles coustani* mosquitoes, sampled in the Negev desert of southern Israel in the early 1980s [7]. EILV grows to high viral titers in insect cells but cannot infect mammalian cells or infant mice [7]. Several barriers of host range restriction of EILV to insects were identified including inhibition of entry and attachment, as well as a restriction of genomic RNA replication [7–9]. The inability of EILV to replicate in mammalian cells has been used to construct a safe to handle CHIKV antigen and a CHIKV vaccine, based on EILV(NSPs)/CHIKV(SPs) chimeric virions [10, 11]. Several other alphaviruses also displaying a host range exclusively restricted to insects were found hereafter, such as the Taï Forest alphavirus (TALV) from Ivory Coast [12], Mwinilunga alphavirus (MWAV) from Zambia [13], Agua Salud alphavirus (ASALV) from Panama [14], and Yada Yada virus (YYV) from Australia [15].

EILV and other insect-specific viruses (ISVs) were shown to affect *in vitro* and *in vivo* a subsequent infection with another virus [16–21]. Such an effect, where a preexisting virus infection impairs a secondary infection with the same (homologous) or with a similar (heterologous) virus is termed superinfection exclusion (SIE) [22, 23]. Prior infection of C7/10 (*Aedes albopictus*) cells with EILV induced both homologous superinfection exclusion in subsequent infections with EILV, as well as heterologous superinfection exclusion in secondary infections with SINV, CHIKV, EEEV, western equine encephalitis virus (WEEV), and Venezuelan equine encephalitis virus (VEEV) [18]. A similar effect was observed *in vivo* for *Aedes aegypti* mosquitoes that were intrathoracic injected with EILV and orally superinfected with CHIKV [18]. Superinfection exclusion has also been observed in other virus families. For example, the insect-specific flavivirus Nhumirim virus was shown to suppress the replication of the mosquito-borne flaviviruses West Nile virus (WNV), Zika virus, Dengue virus, Japanese encephalitis virus, and St. Louis encephalitis virus in C6/36 cells [24–26].

Effects of SIE have been shown at various stages of the viral life cycle, including replication, translation, attachment, and penetration [27–32]. It has recently been shown that the NS2B protein of Japanese encephalitis virus inhibits the replication of Zika virus [33]. It is suggested that SIE in insects might be mediated by viral proteins, such as the alphaviral NSP2 protein, or by the competition for host cell factors, which are necessary in the replication process [32, 34]. Insect-specific viruses can also increase transmission rates of arboviruses in mosquitoes. For example, it was recently shown that two insect-specific viruses block the downregulation of histone H4 in mosquitoes infected with Dengue virus and thereby increased transmission of Dengue from mosquitoes to mice [35]. Thus, insect-specific viruses may modulate vector competence and influence arbovirus transmission dynamics.

So far five human- and animal-pathogenic alphaviruses have been detected in southern Africa, SINV, Middelburg virus (MIDV), CHIKV, Ndumu virus, and Semliki Forest virus [36]. SINV and MIDV seem to be widely distributed in the region [37, 38]. Seroprevalence studies, conducted in the Namibian Zambezi Region in the 1960s, indicated the circulation of CHIKV and SINV [39]. However, only low seroprevalence rates against CHIKV and SINV were found in the local population in the 1980s [40, 41]. Little is known on the diversity and prevalence of alphaviruses in vector populations in southern Africa. In this study we screened mosquitoes from Namibia’s Zambezi Region for alphavirus infection and subsequently studied the effect of a detected insect-specific alphavirus on the replication of endemic vertebrate-pathogenic arboviruses.

## Methods

### Mosquito collection and identification

Permission to conduct fieldwork was granted by the Namibian Ministry of Environment, Forestry and Tourism (MEFT) and the National Commission on Research Science and Technology (NCRST) (permit number RPIV00442018). Mosquitoes were sampled in the north-eastern Namibian Zambezi Region. Sampling was conducted in the Sachinga Livestock Development Centre between May and June 2018, in the Wuparo Conservancy and Mudumu National Park between November and December 2018, in the Mashi Conservancy between March and April 2019 and between February and March 2020, and in the Bwabwata National Park between February and March 2019 [42]. Samples were stored in the field in liquid nitrogen and mosquito species were identified morphologically in the laboratory using standard literature [43–45].

### Viral RNA extraction and PCR screening

Individual mosquitoes were homogenized in phosphate-buffered saline (PBS) using a Tissue Lyser (QIAGEN, Hilden, Germany) system with ceramic beads, and homogenates were arranged into pools of 10 specimens. RNA was extracted with the MagNA Pure 96 DNA and Viral NA Small Volume Kit (Roche Diagnostics, Mannheim, Germany) from pooled mosquito homogenate and subsequently cDNA was synthesized with the SuperScript IV reverse transcriptase kit (Thermo Fisher Scientific GmbH, Dreieich, Germany) using random hexamer primers (Integrated DNA Technologies Germany GmbH, Munich, Germany). Samples were screened for alphavirus infection with a generic PCR assay using a published protocol [12]. Nucleotide sequences, derived by Sanger sequencing (Microsynth Seqlab GmbH, Göttingen, Germany), were analysed in Geneious 9.1.8 (Biomatters Ltd., Auckland, New Zealand) and by using the Basic Local Alignment Search Tool (BLAST) (https://blast.ncbi.nlm.nih.gov/Blast.cgi) of the GenBank database.

### Genome sequencing and analysis

For full genome sequencing, viral RNA was extracted from the infected mosquito or for MP458-NA-2018-PP from infectious cell culture supernatant of C6/36 cells with the QIAzol Lysis Reagent (QIAGEN, Hilden, Germany) and Next generation sequencing (NGS) was performed on a MiSeq desktop sequencer, using the MiSeq Reagent kit v3 (Illumina Inc., San Diego, USA). Assembly of paired end reads was performed in Geneious 9.1.8 (Biomatters Ltd., Auckland, New Zealand). Resulting contigs were mapped to EILV sequences as reference available from GenBank. The virus genomic sequence was confirmed by conventional PCR and Sanger sequencing (Microsynth Seqlab GmbH, Göttingen, Germany). The genomic ends of EILV MP458-NA-2018 were obtained using the 5’-RACE kit (Thermo Fisher Scientific GmbH, Dreieich, Germany). Putative viral open reading frames (ORFs) were identified using Geneious. Transduced amino acid sequences were compared to GenBank using BLASTp.

### Phylogenetic analysis

Nucleotide sequences of viral ORFs were translationally aligned with related alphaviral sequences in Geneious 9.1.8 (Biomatters Ltd., Auckland, New Zealand) using the MAFFT algorithm [46]. The alignment of SPs was based on the E2-6K-E1 polyprotein-ORF and the alignment of NSPs was based on the NSP1-NSP4 ORF, with the NSP3 gene trimmed. All phylogenies were inferred with PhyML, using the Smart Model Selection (SMS) method [47, 48].

### Mosquito species identification by genetic barcoding

A cytochrome c oxidase I (COI) gene fragment was amplified with generic primers for invertebrates and primers designed for *Culex univittatus* mosquitoes (forward 5’-TTAGGAGCTCCAGACATAGCTTTC-3’, reverse 5’-AGGTAATGATAAAAGTAATAAAACAGCAGT-3’) using the alphavirus positive mosquito pool MP458-NA-2018 [49]. PCR products were sequenced by Sanger sequencing (Microsynth Seqlab GmbH, Göttingen, Germany) and compared to the GenBank database using BLAST.

### Virus isolation and plaque purification in cell culture

For virus isolation in cell culture, cells derived from mosquitoes (C6/36, *Aedes albopictus*) and primates (VeroE6, African green monkey kidney) were inoculated with the virus-positive mosquito homogenate MP458-NA-2018 and cultivated for four weeks as previously described [50]. Cell culture supernatants of each passage were checked for virus growth by RT-PCR [12]. A virus stock was generated using the C6/36 cell culture supernatant of the first passage. A plaque purification was performed from the virus stock following established protocols [12, 14]. Another virus stock was prepared from the plaque purified virus and designated MP458-NA-2018-PP. The purity of the MP458-NA-2018-PP stock was confirmed by NGS as described above. A Tissue Culture Infectious Dose 50 (TCID50) end-point dilution assay was used to determine the number of infectious particles for both virus stocks [51].

### Virus growth kinetics

C6/36 cells were infected with MP458-NA-2018-PP in duplicates at a multiplicity of infection (MOI) of 0.1 and incubated at either 28°C, 31°C, or 34°C in order to evaluate the influence of different ambient temperatures on virus replication as described previously [52]. In addition, cells were infected with MP458-NA-2018 in duplicates and incubated at 28°C. Aliquots of cell culture supernatant were taken every 24 hours for five consecutive days and viral genome copy numbers were assessed using qPCR with a plasmid-based standard dilution series as previously described [42]. The qPCR assay was established based on a 184 nucleotide (nt) fragment of the viral RdRP gene (forward 5’-AACCAGCACACATCTACCCA-3’, reverse 5’-TCCGTGTATGATGTTGTCGTC-3’, probe 5’-FAM-CGGTTTGGTGCCATGATGAA-ZEN-3’). Further, cells derived from primates (VeroE6, African green monkey kidney), humans (HEK293T, human kidney), and rodents (BHK-21, hamster kidney) were inoculated with MP458-NA-2018-PP in duplicates at a MOI of 1 and cultivated for four weeks. As control, C6/36 cells were infected with the same inoculum. Cell culture supernatants of each passage were tested for viral replication by qPCR.

### Virus isolates and superinfection experiments

The following virus isolates were used for the superinfection experiments: a CHIKV isolate from the Indian Ocean Lineage [53]; SINV and MIDV were isolated from mosquitoes in Uganda [54]; West Nile virus (WNV) lineage 2 was isolated from a blood donor in Eastern Germany [55]; and Bagaza virus (BAGV) was isolated from mosquitoes collected in Northern Namibia [42]. Except CHIKV, all viruses were isolated from mosquitoes in our laboratory and viral stocks generated from the first or second cell culture passage.

The mosquito cell line C7/10, derived from *Aedes albopictus*, was infected with MP458-NA-2018-PP in duplicates at MOIs of 10 or 1. The cells were additionally either superinfected with SINV, WNV and BAGV in each case at MOIs of 1 and 0.1 and with MIDV at an MOI of 0.1 and with CHIKV at MOIs of 1, 0.1 and 0.01 16 hours post infection with MP458-NA-2018-PP. Aliquots of cell culture supernatants were sampled every 24 hours for four or five consecutive days and numbers of infectious particles were assessed by plaque assay. The entire experiment was repeated in a biological replicate using two technical replicates. Plaque assays were established for each virus using the vertebrate cell lines and overlays indicated in Table 1. Briefly, one day after seeding of cells, cells were inoculated with cell culture samples in serial 10-fold dilutions and incubated for one hour at 37°C and 5% CO_2_. Subsequently the respective overlay medium was added, and cells were incubated between two and four days at 37°C and 5% CO_2_. The overlay was carefully removed, and cells were fixed in 6% paraformaldehyde for 30 minutes, followed by staining for 10 minutes with 0.2% cristal violet.

**Table 1:** Plaque assay conditions for BAGV, CHIKV, MIDV, SINV and WNV.

Virus	Cell line	Format	Cell number/well	Overlay medium	Fixing and staining
BAGV	AGE1.CR	24-well plate	2.4x10^5^	2.5% Avicel, 2x MEM + 10% FCS	4 dpi
CHIKV	VeroFM	12-well plate	2x10^5^	2.5% Avicel, 2x MEM + 5% FCS	4 dpi
MIDV	VeroFM	24-well plate	1x10^5^	2.5% Avicel, 2x MEM + 5% FCS	3 dpi
SINV	VeroE6	24-well plate	2x10^5^	2.5% Avicel, 2x MEM + 5% FCS	2 dpi
WNV	VeroE6	24-well plate	1.8x10^5^	2.5% CMC, 2x MEM + 5% FCS	3 dpi

### Statistical analyses

We used t tests to compare differences in the mean virus replication of the investigated viruses either pre-infected with EILV MP458-NA-2018-PP or not pre-infectetd at the indicated timepoints. Analyses were performed using SPSS (IBM SPSS Statistics 29.0.0.0).

## Results

### Screening for alphaviruses and virus isolation

RT-PCR screening of the 10,206 mosquitoes for alphavirus infection resulted in one positive pool of *Culex univittatus* mosquitoes, MP458-NA-2018, originating from Mudumu National Park. The sequence fragment showed maximum nucleotide identity of 95.5% to the original EILV isolate EO329 from Israel. The mosquito species was identified by morphology and confirmed by genetic barcoding.

MP458-NA-2018 was successfully isolated in C6/36 cells. The virus induced a strong CPE in the first passage at 3 dpi. The obtained isolate was plaque-purified to obtain a pure virus stock. The purified version of the virus (designated MP458-NA-2018-PP) induced small to medium sized plaques at 3 dpi in C6/36 cells.

### Genome sequencing and genetic characterization

**Fig 1:**
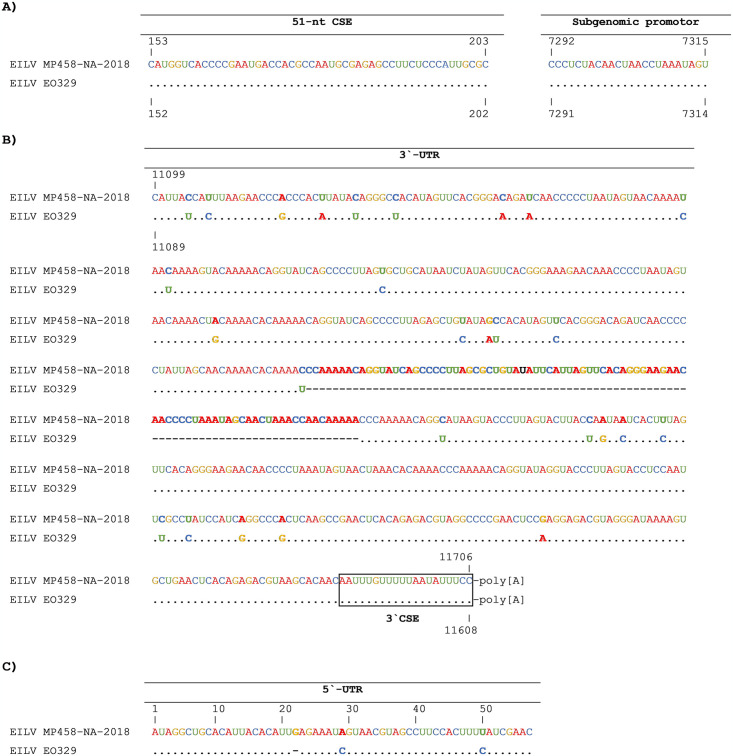
Comparison of selected genome regions of EILV MP458-NA-2018-PP and EILV EO329. Excerpts of selected nucleotide regions of EILV MP458-NA-2018-PP are shown in comparison to EILV EO329. (A) Conserved 51-nt CSE and subgenomic promotor sequence, (B) 5`-UTR (C) 3`-UTR containing the 3`-CSE. Similarities are shown as dots, insertions in bold and deletions with dashed lines.

The genome of MP458-NA-2018 and MP458-NA-2018-PP were sequenced by NGS and the genome termini were determined by RACE-PCR. Analysis of MP458-NA-2018-PP confirmed a pure virus stock, with 100% identity to the wild-type virus sequence. Analysis of the entire genome of MP458-NA-2018 confirmed the first detection of EILV in southern Africa. We observed a genome length of 11,706 nucleotides excluding the 3’-UTR poly(A) tail. MP458-NA-2018 showed the same alphavirus-typical genome organization as EILV isolate EO329 (NC_018615). The NSP- and SP-ORFs of MP458-NA-2018 had pairwise nucleotide identities of 95.5% and 94.9% and pairwise amino acid identities of 97.8% and 97.7% to EILV EO329, respectively, indicating the detection of an EILV variant, tentatively designated EILV MP458-NA-2018. It included three conserved sequence elements (CSEs), which are identical to the ones published for EILV EO329, SP1 CSE (51-nt CSE), the subgenomic promoter CSE, and 3′ CSE (Fig 1A and 1B). The 5’-UTR of EILV MP458-NA-2018 was 57 nt in length and contained two nucleotide exchanges and a single nucleotide insertion when compared to EILV EO329 (Fig 1C). The translated protein ORFs of EILV MP458-NA-2018 contained all protease cleavage sites, as well as the E1 fusion peptide and ribosomal binding site (RBS) motifs postulated for EILV EO329. The 3’-UTR of MP458-NA-2018 was found be 608 nt in length followed by a poly(A) tail in the range from 11-mers to 17-mers. Interestingly, the 3’-UTR of MP458-NA-2018 showed an insertion of 88 nucleotides in its middle part and 27 nucleotide exchanges in comparison to the 3’-UTR of EILV EO329.

### Phylogenetic analysis

**Fig 2:**
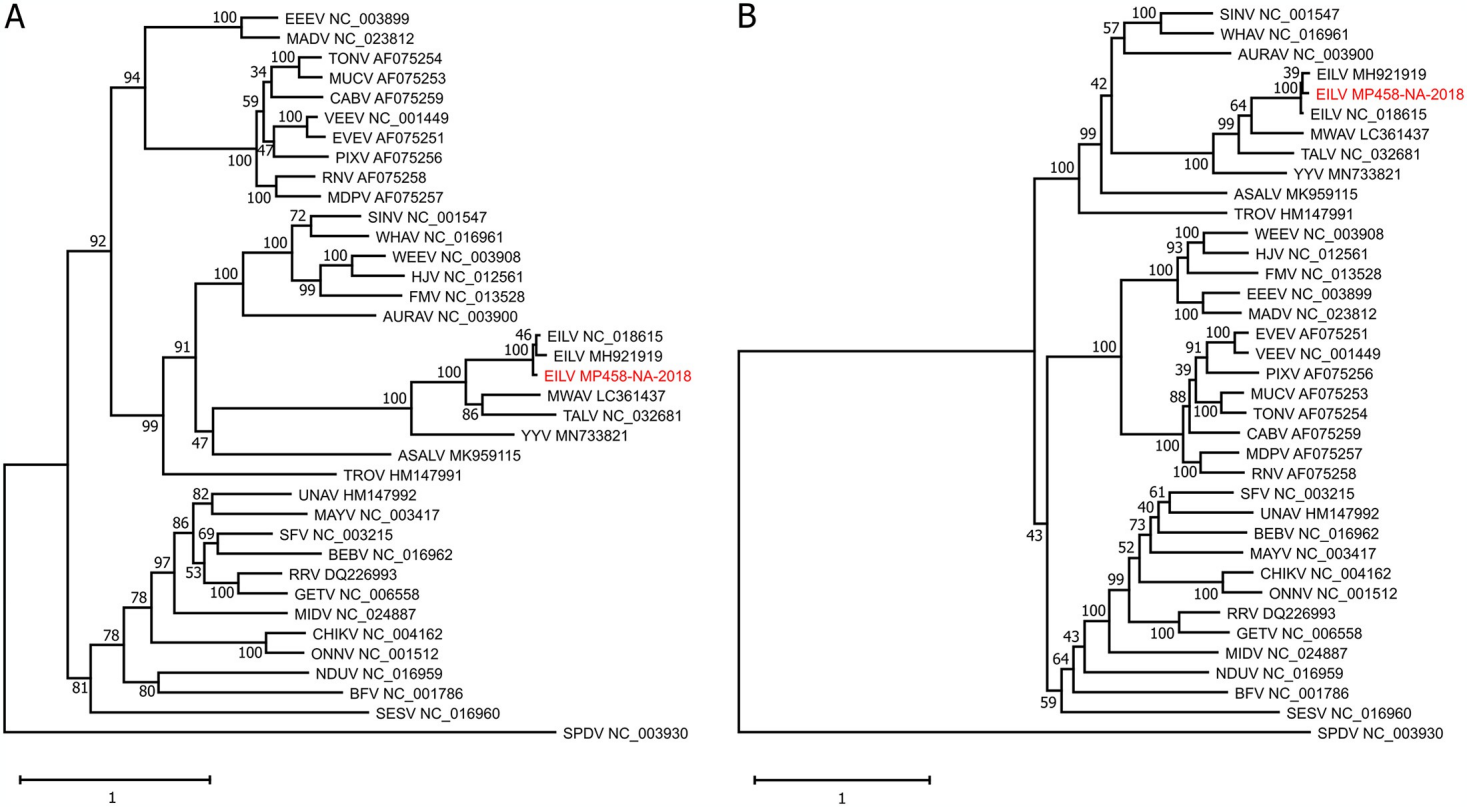
Phylogenetic relationship of the detected EILV strain MP458-NA-2018. Maximum likelihood trees were based on the nucleotide sequences of the viral SP ORF (**A**) and the viral NSP ORF (**B**). The phylogeny based on the viral SPs was calculated using the GTR model with an estimated proportion of invariable sites of 0.09 and estimated gamma shape parameter of 0.712, as chosen by Smart Model Selection in PhyML (SMS). The phylogenetic tree based on the viral NSPs was inferred with the GTR model with an estimated proportion of invariable sites of 0.167 and an estimated gamma shape parameter of 0.591. The phylogenetic trees were rooted on midpoint and confidence testing was performed based on 1,000 bootstrap iterations, as shown for each branch. Virus sequences detected in this work are shown in red. Accession numbers are given for reference sequences accessed from GenBank. Virus name abbreviations are as follows: ASALV, Agua Salud alphavirus; AURAV, Aura virus; BFV, Barmah Forest virus; BEBV, Bebaru virus; CABV, Cabassou virus; CHIKV, Chikungunya virus; EEEV, Eastern equine encephalitis virus; EILV, Eilat virus; EVEV, Everglades virus; FMV, Fort Morgan virus; GETV, Getah virus; HJV, Highlands J virus; MADV, Madariaga virus; MAYV, Mayaro virus; MIDV, Middelburg virus; MDPV, Mosso das Pedras virus; MUCV, Mucambo virus; MWAV, Mwinilunga alphavirus; NDUV, Ndumu virus; ONNV, O’nyong-nyong virus; PIXV, Pixuna virus; RNV, Rio Negro virus; RRV, Ross River virus; SPDV, Salmon pancreas disease virus; SFV, Semliki Forest virus; SINV, Sindbis virus; SESV, Southern elephant seal virus; TALV, Taï Forest alphavirus; TONV, Tonate virus; TROV, Trocara virus; UNAV, Una virus; VEEV, Venezuelan equine encephalitis virus; WEEV, Western equine encephalitis virus; WHAV, Whataroa virus; YYV, Yada Yada virus.

Maximum likelihood phylogenetic analyses based on an alignment of the viral SPs showed that EILV MP458-NA-2018 groups with the EILV strains from Israel and from Morocco (Fig 2A). As observed earlier, the EILV strains formed a distinct clade with the other insect-specific alphaviruses, MWAV, TALV, YYV, and ASALV, in sister relationship to viruses of the Western equine encephalitis (WEE) complex. EILV MP458-NA-2018 also grouped within the ISV alphaviruses in phylogenetic analyses based on the viral NSPs (Fig 2B). However, in this phylogeny the group of ISVs was placed as a sister clade to a clade formed by SINV, Whataroa virus, and Aura virus, with ASALV placed in basal position to both clades as reported previously in other studies [13–15].

### *In vitro* temperature sensibility and host range assessment

**Fig 3:**
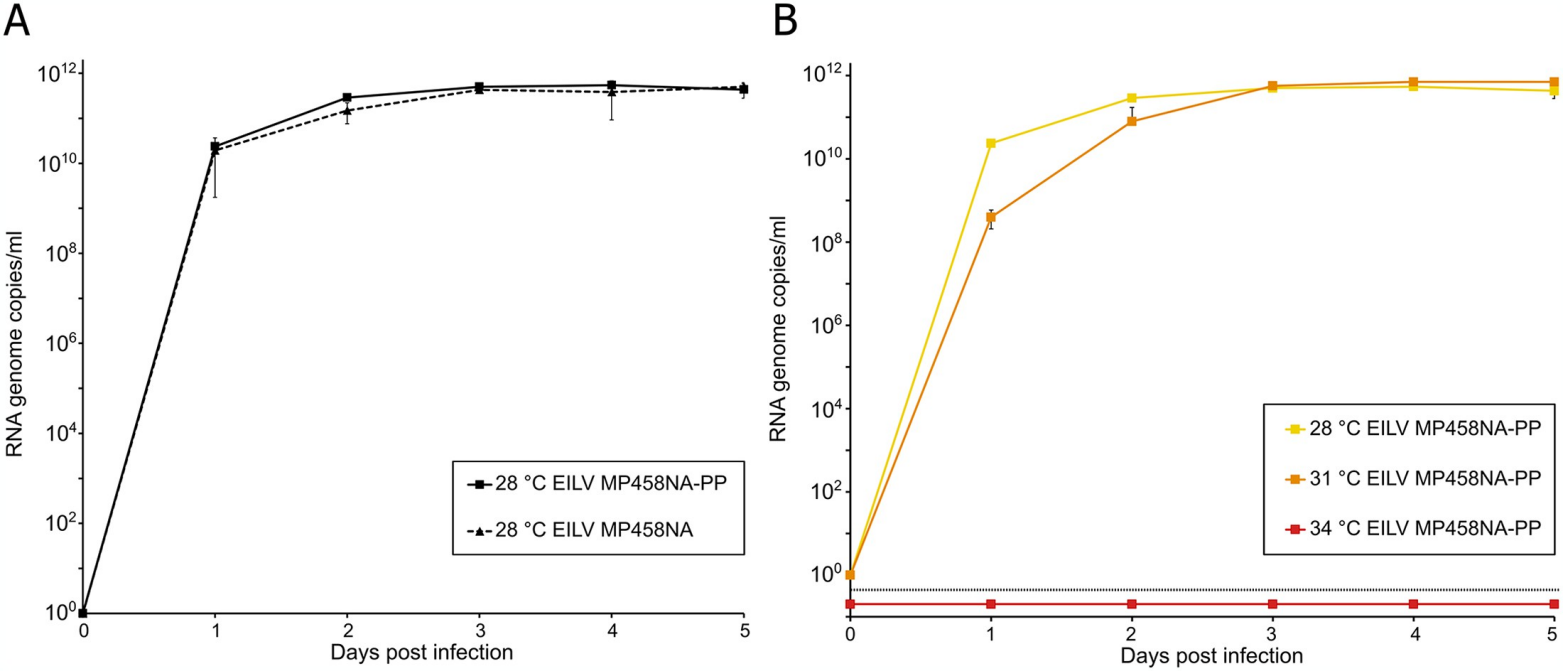
*In vitro* growth analysis and temperature sensitivity of EILV MP458-NA-2018. (**A**) Growth analysis of original and plaque-purified version of EILV MP458-NA-2018. The mosquito cell line C6/36 (derived from *Aedes albopictus*) was infected in duplicates at an MOI of 0.1. A sample of cell culture supernatant was taken every 24 hours for five consecutive days and viral copy numbers were determined by RT-qPCR. (**B**) Temperature sensitivity analysis of EILV MP458-NA-2018-PP. The mosquito cell line C6/36 (derived from *Aedes albopictus*) was infected in duplicates at an MOI of 0.1. Cells were incubated at 28°C, 31°C, or 34°C. A sample of cell culture supernatant was taken every 24 hours for five consecutive days and viral copy numbers were determined by RT-qPCR.

Growth analyses in C6/36 cells showed high genome copy numbers and revealed no fitness impairment of the plaque purified virus (MP458-NA-2018-PP) in comparison to the wildtype (Fig 3A). To assess if the Namibian EILV variant also shows a host range restriction to insects, MP458-NA-2018-PP was inoculated into different vertebrate and insect cells and cultivated for four weeks. Virus replication was detected by RT-qPCR. EILV MP458-NA-2018-PP did not replicate in any of the tested vertebrate cell lines, BHK-21, HEK293T, and VeroE6. To further test if MP458-NA-2018 cannot replicate at vertebrate body temperatures, C6/36 cells were infected with MP458-NA-2018-PP and incubated at different temperatures. Incubation at 31°C slightly impaired virus replication during the first 62 hours post infection (hpi) (Fig 3B). Similar gene copy numbers of 10^11^ copies/ml were reached at 3 dpi as well as when incubated at 28°C. No virus replication was detected at 34°C.

### Superinfection experiments

**Fig 4:**
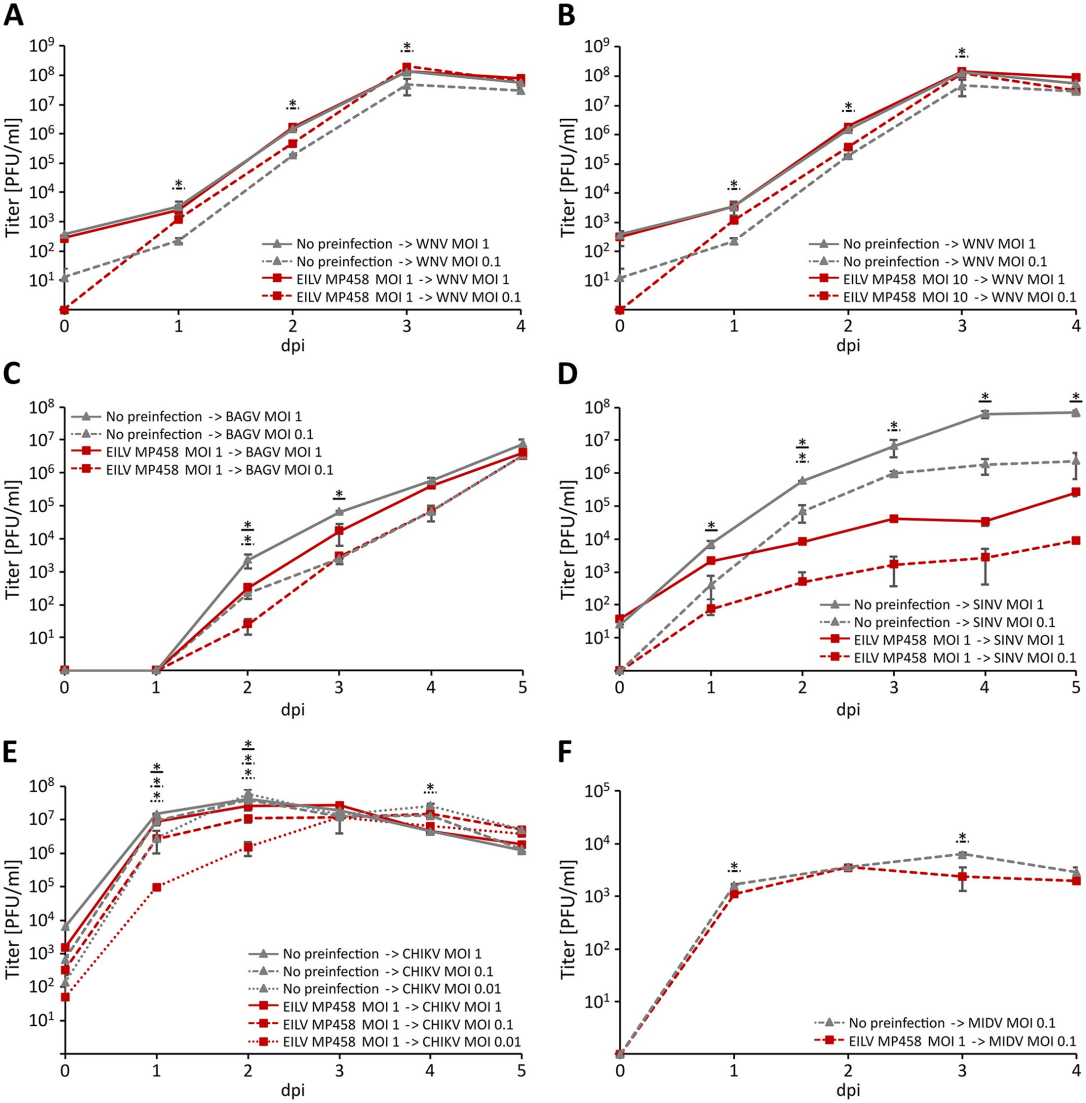
Superinfection experiments with EILV MP458-NA-2018 and different arboviruses. The mosquito cell line C7/10 (derived from *Aedes albopictus*) was infected with EILV MP458-NA-2018-PP in duplicates at the indicated MOIs of 10 and 1. After 16 hours, cells were super-infected with WNV (**A, B**), BAGV (**C**), SINV (**D**), CHIKV (**E**), or MIDV (**F**) at the indicated MOIs. A sample of cell culture supernatant was taken every 24 hours for four or five consecutive days and number of infectious particles were determined by plaques assay. Significant differences in replication of the investigated arboviruses under superinfection were determined by t tests using SPSS and p-values are shown in S1 Table.

To test if the presence of EILV MP458-NA-2018 PP has an effect on superinfecting arboviruses, we selected the three alphaviruses CHIKV, SINV and MIDV that are endemic in southern Africa, the latter two were found to be impaired by EILV in previous studies [18], as well as the two flaviviruses WNV and BAGV that have been detected previously in the same region and same mosquito species [42], for superinfection experiments. Pre-infection of cells with EILV MP458-NA-2018-PP at MOIs of 1 and 10 and superinfection in each case with WNV at MOIs of 1 and 0.1, respectively, did not show any difference between both EILV MOIs on the number of plaque forming units of WNV suggesting that a MOI of 1 was sufficient for a saturating infection with EILV (Fig 4A and 4B; S1 Table). However, in both cases superinfection with WNV at a MOI of 0.1 was slightly enhanced about 6-fold in cells pre-infected with EILV MP458-NA-2018-PP compared to mock infected cells. In contrast, suppression of BAGV titers was found in cells preinfected with EILV MP458-NA-2018-PP, more pronounced in cells superinfected with BAGV at a MOI of 1 than at 0.1 (Fig 4C). A approximately 500- to 2000-fold suppression of infectious particles was found for SINV in cells preinfected with EILV MP458-NA-2018-PP that remained constant over the course of infection with both MOIs (Fig 4D). For CHIKV, we detected minimal suppression of infectious particle production for MOIs of 1 and 0.1 in cells preinfected with EILV MP458-NA-2018-PP, whereas an approximately 10- to 30-fold suppression of CHIKV infectious particles was found at 1 and 2 dpi in cells preinfected with EILV MP458-NA-2018-PP and superinfected with CHIKV at an MOI of 0.01 (Fig 4E). EILV MP458-NA-2018-PP also reduced infectious particle production of MIDV approximately 3-fold at 3 dpi compared to mock infected cells (Fig 4F). All analyses were performed in duplicates and confirmed in a repeated independent experiment.

## Discussion

Alphaviruses with a host range restricted to insects are rarely detected in wild mosquito populations and represent a comparatively small group of viruses when compared to insect-specific viruses of other families. In this study, we detected and characterized an EILV variant isolated from mosquitoes from the Zambezi Region of north-eastern Namibia.

So far, EILV was found in *Anopheles coustani* mosquitoes from the Negev desert in Israel, a stone desert type landscape and in *Culex pipiens* mosquitoes collected in and around the Moroccan capital city of Rabat [7, 56]. In this study, EILV was isolated from *Culex univittatus* mosquitoes originating from Mudumu National Park, a shrub savanna type landscape, which is undisturbed from human cultivation for more than 40 years and is a state protected area [57]. Mosquitoes of the genera *Anopheles*, *Culex*, and *Aedes* have been shown to be susceptible for infection with EILV in laboratory experiments [8]. All EILV detections originate from landscapes with relatively hot and dry climate and a unimodal rainfall pattern [58–60]. We found a low infection rate of 0.098% (1/10,206 mosquitoes positive) which is in agreement with the low detection rates of insect-specific alphaviruses observed in other studies with the exception of EILV detection in Morocco [7, 12–15, 56].

EILV MP458-NA-2018 contained all the alphavirus-typical genetic elements also found in the original EILV strain EO329 from Israel. However, in the 3’-UTR we found a large 88 nt insertion that was not present in EO329. As the 3’-UTR of the Moroccan strain is not complete, we do not know how common such insertions are on EILV. We found poly(A) tails ranging in lengths from 11 to 17 Adenosine residues. From studies with polyadenylated viruses we know that the length of poly(A) tails is not always static but can vary between quasispecies of one virus or between different points in the viral infection cycle [61–64]. Our data confirm the host range restriction of EILV to insects as concluded from previous studies [7, 9]. EILV MP458-NA-2018-PP did not replicate in the tested vertebrate cell lines and its replication was impaired at 31°C and completely blocked at 34°C [7].

ISVs have been shown to interfere *in vitro* and *in vivo* with arboviruses [16–21]. Here, we show that MP458-NA-2018-PP influences the replication of vertebrate-pathogenic alpha- and flaviviruses. We studied the effects of MP458-NA-2018-PP on the alphaviruses CHIKV, MIDV and SINV that are endemic in southern Africa, as well as on the flaviviruses WNV and BAGV that were isolated from the same mosquito vector in the same region [42]. CHIKV and SINV superinfection exclusion had previously been reported in C7/10 cells pre-infected with EILV EO329 [18]. For SINV, a maximal 5,000-fold reduction of infectious particles was observed 1 dpi and no difference to non-pre-infected cells was observed 3 dpi in cells superinfected with EO329 in another study [18]. In our study, we observed only a 500- to 2000-fold reduction in infectious particles that was consistent over the entire course of infection until 5 dpi. EILV EO329 also reduced rates in CHIKV particle production to a higher and to a more stable extend than MP458-NA-2018-PP. However, rate of reduction of CHIKV infectious particle production was similar between our study in cells preinfected with MP458-NA-2018-PP and superinfected with CHIKV at a MOI of 0.01 and the previous study [18]. The observed differences could be due to several reasons. MP458-NA-2018 and EO329 might differ in their phenotypic behavior, as well as different strains of CHIKV and SINV were used that may show difference in their susceptibility to interact with EILV [20, 65]. Effects on EILV on MIDV have not been studied before. Furthermore, we have demonstrated that MP458-NA-2018 also influences infectious particle production of two members of the genus *Flavivirus*, WNV and BAGV, which were previously found to be co-circulating in the Namibian Zambezi Region [42]. For WNV, we found enhanced infectious particle production in cells coinfected with MP458-NA-2018, whereas infectious particle production was approximately 5- to 10-fold suppressed at 2 and 3 dpi compared to mock-infected cells. These data expand our knowledge on SIE of insect-specific alphaviruses to flaviviruses. As WNV and BAGV in the Zambezi Region were both detected in *Culex univittatus* mosquitoes, just like EILV MP458-NA-2018, an interaction of the different virus species in nature is imaginable. Nevertheless, the low EILV infection rate that was found in our study, which is in agreement with low detection rates of other insect-specific alphaviruses in wild mosquito populations [12, 14], suggest that superinfections of mosquitoes infected with EILV and superinfected with a mosquito-borne arbovirus under natural conditions may rarely happen. Further experiments on the interference of EILV with WNV and BAGV, as well as with SINV, CHIKV, and MIDV are needed to assess a possible effect *in vivo*. Although no other alphaviruses apart from EILV were found in this study, seroprevalence studies suggest the presence of SINV, MIDV and CHIKV in the Zambezi Region [39–41].

Future directions to explore the use of ISVs as tools to control transmission of pathogenic arboviruses involve the use of ISVs in natural vector control strategies [66, 67], infection of mosquitoes with specifically engineered ISVs that reduce their fertility or fitness [68, 69], or the development of ISVs as novel vaccine candidates and safe to handle diagnostic antigens enabled by their lack of ability to replicate in vertebrate cells [10, 11, 70–76].

## Supporting information

S1 Table P-values for the growth analyses shown in Fig 4. Statistical differences in viral replication (PFU/ml) between groups were analyzed by t-test for each timepoint using SPSS (IBM SPSS Statistics 29.0.0.0).
(DOCX)
