# Supplementary material for: Eilat virus isolated from Culex univittatus mosquitoes from the Namibian Zambezi Region influences in vitro superinfection with alpha- and flaviviruses in a virus-species-dependent manner
Source: PLoS One. 2024 Dec 20;19(12):e0312182. doi: 10.1371/journal.pone.0312182 (PMC11661592; doi:10.1371/journal.pone.0312182)
Supplement: S1 Table — Statistical differences in viral replication (PFU/ml) between groups were analyzed by t-test for each timepoint using SPSS (IBM SPSS Statistics 29.0.0.0). (DOCX) [file pone.0312182.s001.docx]

**Supporting Information**

**S1 Table:** **P-values for the growth analyses shown in Figure 4.** Statistical differences in viral replication (PFU/ml) between groups were analyzed by t-test for each timepoint using SPSS (IBM SPSS Statistics 29.0.0.0).

| Comparison | Timepoint | p-value |
| --- | --- | --- |
| No preinfection -> WNV MOI 1 vs. EILV MP458 MOI 1 -> WNV MOI 1 | 1 dpi | ns; 0.528 |
|  | 2 dpi | ns; 0.161 |
|  | 3 dpi | ns; 0.580 |
|  | 4 dpi | ns; 0.150 |
| No preinfection -> WNV MOI 0.1 vs. EILV MP458 MOI 1 -> WNV MOI 0.1 | 1 dpi | *; <0.001 |
|  | 2 dpi | *; <0.001 |
|  | 3 dpi | *; <0.001 |
|  | 4 dpi | ns; 0.130 |
| No preinfection -> WNV MOI 1 vs. EILV MP458 MOI 10 -> WNV MOI 1 | 1 dpi | ns; 0.992 |
|  | 2 dpi | ns; 0.090 |
|  | 3 dpi | ns; 0.498 |
|  | 4 dpi | ns; 0.061 |
| No preinfection -> WNV MOI 0.1 vs. EILV MP458 MOI 10 -> WNV MOI 0.1 | 1 dpi | *; <0.001 |
|  | 2 dpi | *; 0.014 |
|  | 3 dpi | *; 0.012 |
|  | 4 dpi | ns; 0.670 |
| No preinfection -> BAGV MOI 1 vs. EILV MP458 MOI 1 -> BAGV MOI 1 | 2 dpi | *; 0.048 |
|  | 3 dpi | *; 0.005 |
|  | 4 dpi | ns; 0.381 |
|  | 5 dpi | ns; 0.343 |
| No preinfection -> BAGV MOI 0.1 vs. EILV MP458 MOI 1 -> BAGV MOI 0.1 | 2 dpi | *; 0.013 |
|  | 3 dpi | ns; 0.494 |
|  | 4 dpi | ns; 1.000 |
|  | 5 dpi | ns; 0.932 |
| No preinfection -> SINV MOI 1 vs. EILV MP458 MOI 1 -> SINV MOI | 1 dpi | *; 0.007 |
|  | 2 dpi | *; 0.004 |
|  | 3 dpi | ns; 0.052 |
|  | 4 dpi | *; 0.007 |
|  | 5 dpi | *; 0.006 |
| No preinfection -> SINV MOI 0.1 vs. EILV MP458 MOI 1 -> SINV MOI 0.1 | 1 dpi | ns; 0.249 |
|  | 2 dpi | *; 0.048 |
|  | 3 dpi | *; 0.002 |
|  | 4 dpi | ns; 0.062 |
|  | 5 dpi | ns; 0.166 |
| No preinfection -> CHIKV MOI 1 vs. EILV MP458 MOI 1 -> CHIKV MOI | 1 dpi | *; 0.015 |
|  | 2 dpi | *; 0.023 |
|  | 3 dpi | ns; 0.372 |
|  | 4 dpi | ns; 0.766 |
|  | 5 dpi | ns; 0.537 |
| No preinfection -> CHIKV MOI 0.1 vs. EILV MP458 MOI 1 -> CHIKV MOI 0.1 | 1 dpi | *; 0.008 |
|  | 2 dpi | *; 0.003 |
|  | 3 dpi | ns; 0.775 |
|  | 4 dpi | ns; 0.637 |
|  | 5 dpi | ns; 0.085 |
| No preinfection -> CHIKV MOI 0.01 vs. EILV MP458 MOI 1 -> CHIKV MOI 0.01 | 1 dpi | *; 0.041 |
|  | 2 dpi | *; 0.015 |
|  | 3 dpi | ns; 0.742 |
|  | 4 dpi | *; 0.004 |
|  | 5 dpi | ns; 0.387 |
| No preinfection -> MIDV MOI 0.1 vs. EILV MP458 MOI 1 -> MIDV MOI 0.1 | 1 dpi | *; 0.011 |
|  | 2 dpi | ns; 0.982 |
|  | 3 dpi | *; 0.012 |
|  | 4 dpi | ns; 0.301 |
